# Leptomeningeal Relapse of Acute Promyelocytic Leukemia

**DOI:** 10.14740/wjon761w

**Published:** 2014-05-06

**Authors:** Tarik Hadid, Salman Fazal, John Lister

**Affiliations:** a Van Elslander Cancer Center, St. John Providence Health System, Grosse Pointe Woods, Michigan, USA; b Division of Hematology and Cellular Therapy, Western Pennsylvania Cancer Institute, West Penn Allegheny Health System, Pittsburgh, Pennsylvania, USA

**Keywords:** Acute promyelocytic leukemia, Extramedullary relapse, Leptomeningeal relapse

## Abstract

Extramedullary relapse (EMR) of acute promyelocytic leukemia (APL) is a rare entity, with predilection to involve the central nervous system (CNS). Risk factors include leukocytosis of > 10 × 10^9^/L, bcr3 isoform, microgranular variant, age > 45 years and development of subarachnoid hemorrhage (SAH) during induction therapy. We report a case of APL who completed induction and consolidation therapy but subsequently relapsed with leptomeningeal involvement. Retrospectively, we identified several risk factors for EMR in our patient. Interestingly, the use of all-trans retinoic acid has recently been associated with higher risk of EMR possibly due to up-regulation of adhesion molecules on the surface of the leukemic cell, resulting in their passage through the endothelium to extramedullary tissues. However, data remain conflicting in that regard. Although universal CNS prophylaxis has been suggested, the low incidence of EMR among APL patients renders this strategy less attractive. Nonetheless, active surveillance and CNS prophylaxis may be considered in patients at high risk for EMR, particularly in those of SAH during induction therapy. Further research is needed to evaluate the effectiveness and safety of this strategy.

## Introduction

Acute promyelocytic leukemia (APL) is a highly curable myeloid neoplasm with the majority of failures attributed to early hemorrhagic complications [1, 2]. Extramedullary relapse (EMR) with or without concomitant medullary relapse has rarely been described in the medical literature. In this report, we discuss a case of APL with central nervous system (CNS) relapse that occurred 2 months after completion of consolidation therapy. We also review various risk factors for EMR and propose a surveillance strategy to be considered in patients at high risk for EMR.

## Case Report

**Figure 1:**
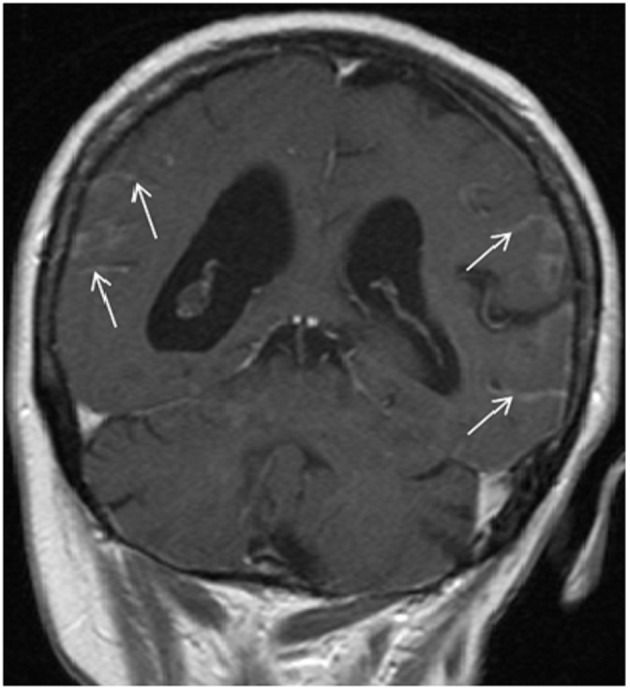
T1-weighted MRI image showing leptomeningeal enhancement (arrows).

**Figure 2:**
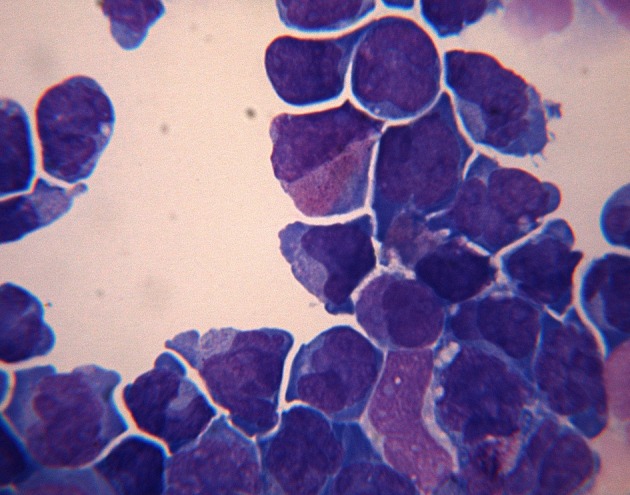
Cerebrospinal fluid is hypercelluar with numerous hypogranular promyelocytes.

A 66-year-old woman presented with a 2 weeks history of fever, exertional dyspnea and generalized weakness. Physical examination was unremarkable except for temperature of 38.7°C and scattered ecchymoses. Complete blood count revealed WBC of 26.5 × 10^9^/L with 75% blasts, hemoglobin of 9.1 g/dL, platelet of 15 × 10^9^/L and lactate dehydrogenase (LDH) of 2,039 IU/L. Peripheral smear revealed immature cells with irregular, lobulated nuclear contour, scant cytoplasm, fine granules and occasional Auer rods. Bone marrow aspiration and biopsy confirmed the diagnosis of microgranular variant of APL. Cytogenetic and molecular testing detected the presence of bcr3 of PML-RARα consistent with t(15;17)(q22;q21). She was initiated on induction therapy with all-trans retinoic acid (ATRA) 45 mg/m^2^ twice daily until complete remission (CR) and idarubicin 12 mg/m^2^ daily on day 2, 4, 6 and 8 [3]. Unfortunately, treatment course was complicated by subarachnoid hemorrhage (SAH) and severe differentiation syndrome requiring mechanical ventilation and interruption of therapy on day 5. She subsequently recovered and resumed induction therapy with single agent ATRA and achieved complete cytogenetic and hematological remission but molecular remission was not confirmed. Due to a poor performance status, she was not a candidate for consolidation chemotherapy and therefore, received consolidation therapy with ATRA for 8 cycles and arsenic trioxide (ATO) for 4 cycles [4]. Two months after completion of therapy, she developed progressive headaches and severe photophobia. At that time, WBC was 6.6 × 10^9^/L with no abnormal cells, hemoglobin of 12.3 g/dL, platelet of 227 × 10^9^/L and LDH of 146 IU/L. Magnetic resonance imaging (MRI) of the brain showed extensive leptomeningeal enhancement (Fig. 1). Cerebrospinal fluid (CSF) was very cellular with many immature cells with morphology similar to that seen in the bone marrow at initial diagnosis (Fig. 2). Additionally, flow cytometry detected a similar immunophenotype to that noted at initial diagnosis. Despite continued hematological and cytogenetic remission, reverse transcription polymerase chain reaction of peripheral blood detected the brc3 isoform of PML-RARα confirming molecular relapse. She was treated with repeated courses of intrathecal methotrexate and cytarabine. With therapy, she showed clinical improvement and resolution of her symptoms with clearance of her CSF. Systemic therapy was deferred to the outpatient setting given her poor performance status. She was non-compliant and lost to follow-up.

## Discussion

EMR, as in our patient, is rarely reported in patients treated for APL [5, 6]. EMR can occur in the CNS [5-11], skin [5, 6, 9, 12], middle ear [5, 9, 11, 13], lung [5], pleura [14], sites of vascular access [12] and sites of bone marrow aspiration and biopsy [12]. However, CNS is the predominate site of EMR with an estimated risk of 0.6 to 2% [5, 6]. Several risk factors for EMR have been identified. One European study found leukocytosis (> 10 × 10^9^/L), bcr3 isoform of PML-RARα and younger age (< 45 years) to be significantly associated with higher risk for EMR. However, only leukocytosis was found to be significant on multivariate analysis [6]. In addition, SAH during induction therapy and microgranular variant were found to portend higher risk of EMR in other studies [15-17]. In fact, the probability of EMR approaches 19% in those with SAH compared to 1% in those without SAH [17]. This may be due to leukemic cells seeding the leptomeninges when SAH occurs. Notably, our patient has multiple risk factors including leukocytosis, bcr3 isoform, microgranular variant and SAH during induction therapy, which placed her at significant risk for CNS relapse.

Within the last two decades, there has been significant concern regarding the association between the use of ATRA and the emergence of EMR. It has been hypothesized that ATRA may up-regulate cellular adhesion molecules expressed on the surface of leukemic cells such as CD11a, CD11b, CD11c and CD45RO. Binding of these molecules to their ligands expressed on endothelial cells may result in passage of these cells through the endothelium, which results in EMR [8, 15, 18, 19]. To test this hypothesis, Specchia et al reviewed the outcome of two consecutive studies of the "Gruppo Italiano Malatie Ematologiche dell’Adulto" and found no significant association between ATRA and EMR. However, there was a worrisome trend toward higher incidence of EMR among the ATRA group (12% versus 5%, P = 0.08). Furthermore, the pattern of EMR was different between the two cohorts with those who received ATRA suffering higher CNS relapses compared to the chemotherapy-only cohort (8% versus 1%, P = 0.02) [5]. Therefore, CNS relapse remains a concern in patients who receive ATRA, particularly in the presence of other risk factors, as in our patient. Whether these findings represent a true association or merely a consequence of improved survival of APL patients remains to be established.

There is no consensus on the best strategy to treat EMR. The majority of reported cases were treated with intrathecal chemotherapy with or without radiation therapy [6, 8, 10]. ATRA, ATO and/or chemotherapy were also used to treat systemic disease when present simultaneously [11, 13, 19-21]. Unfortunately, ATRA has limited penetration to CNS and therefore, is used primarily to treat systemic disease [22]. In addition, no CR was observed with ATRA in relapses occurring within 4 months of discontinuation of ATRA, as in our patient [23]. Conversely, ATO efficiently penetrates the CSF and achieves levels that correlate linearly with and approach 30-50% of the plasma levels [24, 25]. Although these findings suggest that this agent might effectively treat and prevent CNS relapse, its occurrence in our patient after receiving four 8-week cycles of ATO argues otherwise. Systemic ATO might be inadequate for prophylaxis for CNS relapse. Several authors advocate routine intrathecal chemotherapy for CNS prophylaxis in high-risk APL patients [26]. However, the low incidence of CNS relapse argues against this strategy. Nonetheless, selective surveillance and prophylaxis of individuals with high and/or multiple risk factors for CNS relapse after achieving CR may be more appropriate in successfully preventing CNS relapse while avoiding unnecessary risky interventions. Further study of surveillance and prophylaxis strategy to prevent CNS relapse in APL patients is needed to standardize therapy for this increasingly curable disease.
